# Evaluation of Nrf2, Keap1 and Apoptotic Pathway Genes Expression in Acute Myeloid Leukemia Patients

**DOI:** 10.22037/ijpr.2019.14907.12738

**Published:** 2021

**Authors:** Amir Jalali, Sara Mahmoudi, Amir Larki Harchegani, Javad Mohammadiasl, Ahmad Ahmadzadeh

**Affiliations:** a *Department of Toxicology, School of Pharmacy and Toxicology Research Center, Ahvaz Jundishapur University of Medical Sciences, Ahvaz, Iran. *; b *Department of Pharmacology and Toxicology, School of Pharmacy, Hamadan University of Medical Sciences, Hamadan, Iran.*; c *Department of Medical Genetics, School of Medicine, Ahvaz Jundishapur University of Medical Sciences, Ahvaz, Iran. *; d *Health Research Institute, Research Center of Thalassemia and Hemoglobinopathy, Ahvaz Jundishapur University of Medical Sciences, Ahvaz, Iran. *; e *Department of Operating Room, School of Paramedical Sciences, Guilan University of Medical Sciences, Rasht, Iran.*

**Keywords:** Nrf2, Bcl-2, Bcl-XL, Keap1, Acute Myeloid Leukemia, Real-Time PCR

## Abstract

The aim of this study was to evaluate the expression Nrf2 (Nuclear factor-erythroid 2-p45 derived factor 2) and Keap1 (Kelch-like ECH-associated protein 1) genes and Bcl-2 (B-cell lymphoma 2), Bcl-XL (B-cell lymphoma-extra large), Bax (Bcl2-associated X protein) apoptotic pathway genes in acute myeloid leukemia patients. In this case-control study, the expression of genes encoding Nrf2, Keap1, Bcl2, Bcl- XL and Bax in 40 acute myeloid leukemia (AML) patients were compared with 40 normal individuals in the Iranian population. We evaluated the mRNA expression of genes by using the real-time quantitative polymerase chain reaction. The expression of Nrf2, Bcl2 and Bcl- XL genes in new AML patients were increased (*p < *0.05). The patients treated with chemotherapy had a significantly more than four times higher expression level of Nrf2 than new case patients (*P < *0.05), while there was a decrease in the expression level of Bcl2 and Bcl-XL, which was not statistically significant. In other hands in relapsed patients, the expressions of Nrf2, Bcl2 and Bcl- XL were higher level than new case patients (*p < *0.05) but this was less than patients treated with chemotherapy (*p > *0.05). The high levels of mentioned genes may be associated with poor treatment response, chemoresistance and disease recurrence. Because of hyperactivation and overexpression of Nrf2 in leukemia, suggest that Nrf2 inhibitors could be used as a pharmacological target in combination with classical chemotherapeutic agents to increase the efficacy of anticancer therapy.

## Introduction

 Acute myeloid leukemia (AML) is a malignant disease of the bone marrow, which hematopoietic precursors are arrested and leads to proliferation and accumulation of abnormal myeloblasts in the bone marrow and peripheral blood (1). It seems genetic and environmental factors like genetic mutations in blood progenitor cells, exposure to some substances such as benzene, ionizing radiation, chemotherapy and immune system disorders may increase the risk of leukemia (2). Several factors such as biological and clinical factors, age, cytogenetic agent and drug resistance affect the treatment outcomes (3). Despite progress in the treatment of acute myeloid leukemia in recent years, resistance to chemotherapy drugs, is a major problem in the treatment of AML.

Nrf2, a transcription factor of the CNC (“cap ‘n’ collar”) family, is a master regulator of cellular detoxification responses and oxidative states. In cancer cells, the Nrf2 pathway is one of the cell survival pathways that protect cells from apoptosis (4). Keap1 negatively regulates Nrf2. Keap1 keeps the expression of Nrf2 in constitutive low level. Under the homeostatic condition, Nrf2 locates in the cytoplasm and binds to Keap1. This complex is a substrate of Cullin3 (Cul3)-dependent E3 ubiquitin ligases. The performance of this ligase leads to ubiquitination and proteasomal degradation of the Nrf2-Keap1 complex (5). In response to oxidative stress or electrophilic compounds, E3 ubiquitin ligase activity is inhibited. Then Nrf2 translocates into the nucleus where it interacts with a small Maf protein (sMaf) and binds to the antioxidant response element (ARE)-dependent cytoprotective genes. This process leads to overexpression of several genes such as antioxidant genes, anti-apoptotic genes, metabolic genes, cytoprotective and detoxifying genes (6). The target genes of Nrf2 play a critical role in cancer cell growth, cell survival and tumorigenesis. The permanent accumulation of Nrf2 or constitutive activation of the Nrf2 pathway signals in cancer cells leads to proliferation, evading from apoptosis, metastasis and drug resistance (7, 8).

The family of B-cell lymphoma 2 (Bcl-2) proteins plays an essential role in the regulation of apoptosis and cell survival. This family divided into pro-apoptotic protein like Bcl2 associated x protein (Bax) and anti-apoptotic proteins, including Bcl2 and B-cell lymphoma-extra-large (Bcl-XL). The high expression of Bcl2 is usually associated with poor cell survival in many human cancers. The expression of the apoptotic pathway genes may be changed by interactions with other cellular signaling proteins. Therefore, each of the interactions was separately involved in determining the final phenotype of cancer cells and response to treatment (9). 

In the present study, we evaluated the alterations in the expression of Nrf2 and Keap1 genes and Bcl-2, Bcl-XL and Bax genes as apoptotic pathway genes in various stages of the disease in patients with AML. The results of this study may be able to demonstrate the effectiveness of chemotherapy in patients with leukemia and the likelihood of drug resistance.

## Experimental

*Study population*

Among the patients referred to the Cancer and Oncology Hospital in Ahvaz (Shafa hospital), a total number of 40 patients (20 females and 20 males) at different stages of AML as well as 40 healthy subjects as a control group were included in the present case-control study. Patients participating in the study were in different stages of treatment (Table 1). These included a number of new cases with definitive diagnosis of the disease, a group of patients under intensive-chemotherapy and finally, patients with AML who after complete remission, again develop recurrent disease are described as recurrence patients. Treatment of AML usually begins with intensive induction chemotherapy, with the aim of achieving a complete remission. Induction chemotherapy usually includes a 7-day continuous infusion of cytarabine accompanied by anthracycline treatment (bolus or short infusion) on days 1-3 (“7+3” regimens). This usually requires hospitalization for at least 4 weeks, waiting for recovery of normal blood count and management of the complications of the disease and chemotherapy adverse effects.

The AML patients were diagnosed according to standard clinical and morphological criteria (the European Leukemia Net (ELN) recommendations for diagnosis and management of AML) by a specialist in oncology.

The study has been approved by the Ethics Committee of Ahvaz Jundishapur University of Medical Sciences (approval number: IR.AJUMS.REC.1393.381). Also, the consent form approved by the Ethics Committee of Ahvaz Jundishapur University of Medical Sciences was signed by the patients participating in this research project.

*RNA extraction*

Peripheral blood sampling was obtained from the population of patients and control subjects. At a time interval of 24 hours after entering the study, 2 ml of venous blood sample from these subjects was added to 15 ml Falcon tubes containing 100 μl of antagonist EDTA at a concentration of 10% and the RNA extraction steps were performed. Total RNA was extracted by RNA extraction kit (Sinaclon, Iran) according to the manufacturer’s instruction. The quality and quantity of extracted RNA were evaluated by 1% agarose gel electrophoresis and total RNA concentration was measured using a BioPhotometer (Eppendorf AG, Hamburg, Germany).

*cDNA synthesis*

The cDNA was synthesized by RevertAid First Strand cDNA Synthesis Kit (Thermo Fisher Scientific, USA) according to the manufacturer’s instructions. Oligo (dT) primer was mixed with template RNA and was incubated at 65˚C for 5 min. Transcriptase reaction buffer, transcriptase, deoxynucleotide mix, RNase inhibitor were added to the previous mixture. Total mixture was incubated at 42˚C for 60 min, and 70˚C for 5 min. The synthesized cDNA was stored at -70˚C.

*Real-time quantitative PCR*

The quantitative real-time PCR reactions and fluorescence measurements were performed by StepOne™ Real-Time PCR System from Applied Biosystems (Thermo Fisher Scientific, USA). The SYBR Green master mix (Qiagen, Hilden, Germany) was used for quantitative analysis and GAPDH (Glyceraldehyde 3-phosphate dehydrogenase) was used as internal control. All reactions were performed in duplicate with appropriate negative controls. The PCR reactions were carried out as follows: Initial denaturation at 95˚C for 10 min; 35 cycles of annealing at 95˚C for 15 sec; extension at 60˚C for 60 sec. Cycle threshold (CT) values of each gene were measured for quantitative analysis. The relative expression of genes was obtained by calculation of ΔCT and 2^- ΔCT^ based on a comparison between the CT value of target genes and the internal control gene. The primers used are shown in Table 2. 

*Statistical analysis*

REST software 2009 as a standalone software tool was used for analysis of gene expression data from quantitative real-time PCR experiments. IBM SPSS statistics software (version 22) and Microsoft Excel were used for statistical calculations. Quantitative data were presented as the mean ± standard error of the mean (SEM). The independent-samples t-test was used to calculate statistical differences between the means of target gene expression in AML patients and the control group. The correlation between the Nrf2 gene and Bcl2, Bcl-xL genes expression were evaluated by Pearson’s Correlation method. P-values of 0.05 or less were considered statistically significant.

## Results

*Expression of Nrf2, Keap1, Bax, Bcl2 and Bcl-XL genes*

The relative expression of Nrf2 gene (7.1 ± 1.3), significantly increased (*p < *0.05) in AML patients (n = 40) in comparison to control group (n = 40). However, there was no significant difference (*p > *0.05) in the relative expression of the Keap1 gene between AML patient (1.2 ± 0.04) and the Control group. The relative expression of the Bcl2 gene in AML patient (5.7 ± 1.3), significantly increased compared to the control group (*p < *0.05). The relative expression of Bcl-XL gene in AML patient (9.6 ± 1.3), significantly increased compared with control values (*p < *0.05). There was no significant difference in the relative expression of the Bax gene between AML patient and control group values (0.9 ± 1.3; *p > *0.05; Tables 3, 4)

*The effect of age and gender on gene expression in AML patients*

 The expression of Nrf2 and Keap1 genes in different age groups of patients showed no statistically significant difference (*P > *0.05) (Table 3). Also, statistical analysis of gene expression data Bcl2, Bcl-XL and Bax genes showed that there were no significant differences (*p > *0.05) among different age groups (Table 4). In addition, no significant difference was observed in the expression of Nrf2, Keap1, Bcl2, Bcl-xl and Bax genes between male (n = 23) and female gender (n = 17) (*p > *0.05).

*The expression of genes in different treatment status*

Comparison of the relative expression of Nrf2 gene in new case patients (NC) (n = 6) (2.13 ± 0.97) and chemotherapy patients (CHT) (n = 30) (8.32 ± 6.23), indicates that there is a significant difference between the two conditions (*P < *0.05), but there is no significant difference among chemotherapy patients (CHT) (n = 30) (8.32 ± 6.23) and recurrence patients (REC) (n = 4) (11.95 ± 8.4) (*p > *0.05). The expression of the Bcl2 gene in NC (11.23 ± 7.94) decreased compared to CHT (5.81 ± 3.8), but this reduction was not statistically significant (*p > *0.05). Also, the expression of Bcl2 gene in the REC (12.83 ± 8.79) increased compared with CHT (5.81 ± 3.8), but there was no statistically significant difference (*P > *0.05). 

**Figure 1:**
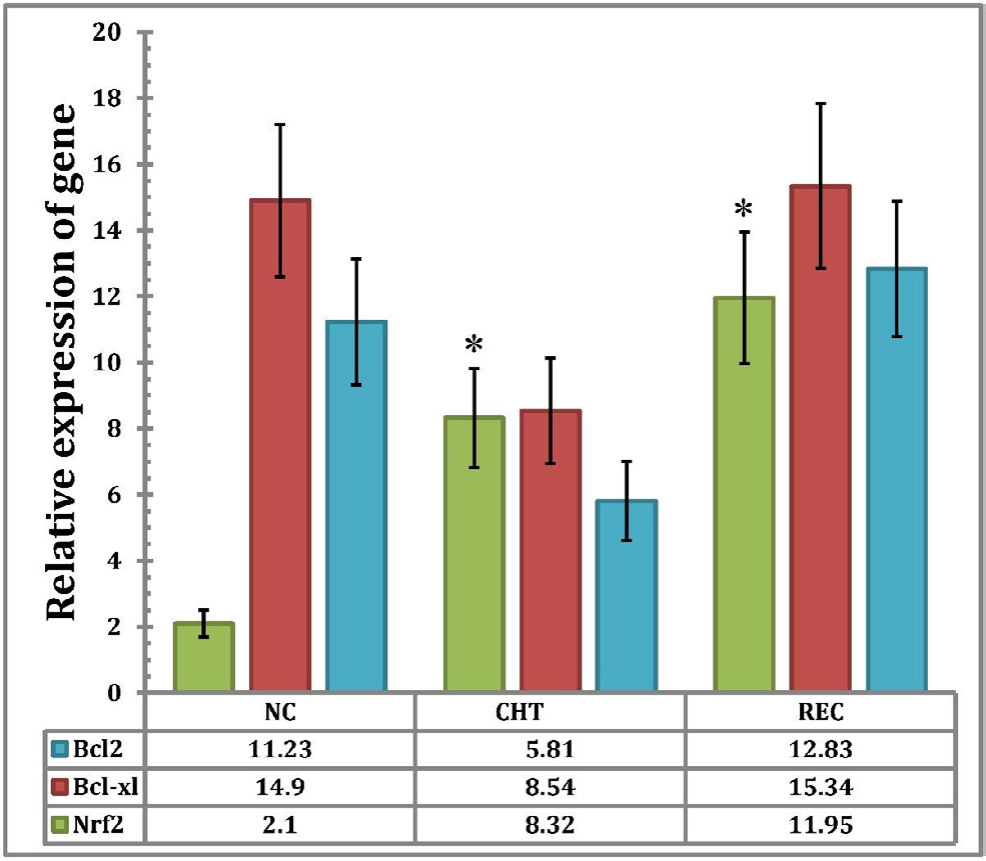
Relative expression of BCL2, BCL-XL and NRF2 genes in AML patients in different treatment status, Statistical analysis of the data was performed by comparing the means through one-way ANOVA and P values ≤ 0.05 were considered as statistically significant. Data expressed as Mean ± SEM. An asterisk sign (*) indicates that the differences in the expression levels of the corresponding genes in NC patients compared to chemotherapy patients and/or recurrence patients are significant). NC = new case Patients; CHT = chemotherapy patients; REC = recurrence patients

The relative expression of Bcl-XL gene in CHT patients (8.54 ± 5.02) decreased compared to NC patients (14.9 ± 8.99) but there was no statistically significant difference between the two groups (*P > *0.05). While the overexpression of the Bcl-XL gene observed in REC patients (15.34 ± 9.15) compared to CHT was not significant (*p >*0.05) (Figure 1).

*Gene expression correlation analysis of Nrf2 related to Bcl2, Bcl-xl and Bax*

Pearson’s method (Pearson’s correlation coefficient) was used to assess the expression of the Nrf2 gene in relation to the expression of Bcl2 and Bcl-XL genes. The results showed that the relative expression level of the Nrf2 gene was significantly correlated with the expression of Bcl2 and Bcl-XL genes (*p < *0.001) (Table 5). 

## Discussion

Currently, achieving an appropriate treatment response is a major challenge in the treatment of many cancers. Despite new medications, chemoresistance is one of the most important causes of treatment failure. Apoptosis is a critical process that its dysregulations results in tumorigenesis and chemoresistance. Recent studies have been shown that transcription factor Nrf2 is highly expressed in various types of cancers. Overexpression of Nrf2 and target genes have been reported in lung, breast, head and neck, ovarian, and endometrial cancers (10-12). Many studies reported the association between elevated levels of Nrf2 and chemoresistance in many cancers (13, 14). The patients with a high expression of Nrf2 have not good prognosis in the process of treatment (15, 16). The overexpression of Nrf2 with an increased level of target genes such as detoxifying enzymes, antioxidants and drug transporting proteins in cancer cells have been shown to promote chemoresistance and radioresistance (17, 18). 

The control mechanism of the Nrf2 gene expression and translocation to the cell nucleus in AML is currently unknown. In non-AML cells, Keap1 as the Nrf2 inhibitor is responsible for the Nrf2 degradation mediated by the ubiquitin-26S proteasome system. KEAP1 in the cytoplasm Upon exposure to stress conditions (electrophilic or oxidative stress), Like the production of reactive oxygen species (ROS) after receiving chemotherapy drugs, (19, 20) Disrupts the proteasomal degradation of Nrf2 caused by Keap1, results its activation and nuclear localization (21), where Nrf2 forms a heterodimer complex with small Maf (sMaf) proteins, that binds to the antioxidant response element (ARE), which results in the activation of ARE-mediated Nrf2-inducible genes expression.

In agreement with the above-mentioned reports, the present study showed that overexpression of Nrf2 was observed in the patients’ group in comparison with the control group, while no significant relevance was observed in Keap1 gene expression. In NC patients the relative expression of the Nrf2 gene was higher than the control group. In patients who were under treatment with CHT, the expression of the Nrf2 gene was higher than NC patients. It seems that the expression of the Nrf2 gene during the early stages of the disease increased but during chemotherapy, extremely increased and leads to Nrf2 overexpression. Many Clinical and experimental studies were demonstrated that Nrf2 overexpression could positively related to tumor progression and chemoresistance in leukemia patients (22). Despite the existence of new treatment strategies like proteasome inhibitors such as daunorubicin, cytarabine and imatinib, constitutive activation of Nrf2 lead to overexpression of drug efflux pumps proteins, detoxification phase II enzymes and antioxidants. Finally, constitutive expression of cytoprotective and detoxification genes, due to constitutive activation of Nrf2 provides resistance to apoptosis and chemoresistance during therapy (23). High expression of Nrf2 in human cancer cells can due to somatic mutations in Keap1 or Nrf2, epigenetic silencing of Keap1, transcriptional upregulation of oncogene signaling related to Nrf2 and aggregation of Nrf2/Keap1 complex disrupting proteins (24). To date, various mutations have been identified in Keap1 and Nrf2 genes in different types of human cancers. Most of these mutations lead to continuous activation of Nrf2 and constitutive expression of cytoprotective genes (25). However, past studies showed that high expression of the Nrf2 gene in AML patients was not associated with any types of mutations in Nrf2 or Keap1 genes (22, 26).

In addition to the upregulation of cytoprotective genes, constitutive expression of Nrf2 can lead to disruption of the main apoptotic pathways. Nrf2 activation inhibited apoptosis and therefore increase cancer cells survival and drug resistance. Recent studies showed that Nrf2 directly activate the transcription of the anti-apoptotic gene like Bcl2 and Bcl-XL (27, 28). In this study, we investigated the expression of Nrf2, Keap1, Bcl2, Bcl-XL and Bax in AML patients. Our results showed that in NC patients, the expressions of the Bcl2 and Bcl-XL genes were higher than the control group. With the starting of treatment due to the effect of chemotherapy the expression of Bcl2 and Bcl-XL genes were decreased, but Nrf2 expression was increased. In NC and CHT patients the Bax gene was slightly expressed. The high level of Bcl2 reduced the formation of Bax/Bcl2 heterodimers. Thus the ratio of Bax/Bcl2 as a parameter for the assessment of apoptosis was decreased and blocked the apoptotic pathways. Therefore, the aforementioned changes in Bcl2 gene expression are causing cancer progression and resistance to chemotherapy. Since in CHT patients, the Nrf2 gene is highly expressed, it can be assumed that altered expression of Bcl2 and Bcl-xl, without a significant increase in expression level of Bax gene and following block of apoptotic pathways, may be due to the influence of Nrf2 on the expression of apoptotic pathway genes. Our Study results showed a statistically significant correlation between Nrf2 expression and Bcl2 and Bcl-XL expressions, which can confirm the aforementioned results.

In agreement with the present results, a study was performed on five different cell lines. The results of this study demonstrated that Nrf2 binds to antioxidant response element (ARE) in Bcl2 and Bcl-XL gene’s promoters and controlled the expression of anti-apoptotic Bcl2 and Bcl- XL genes and cellular apoptosis. Overexpression of Nrf2 upregulates anti-apoptotic Bcl2 and Bcl-XL gene’s expression. Subsequently, up-regulation of Bcl2 and Bcl-XL by Nrf2 activation down-regulate Bax and decrease caspase 3 and 7 activities. This process prevents apoptosis and leads to drug resistance (27, 28).

In another study by Jaiswal and et al, it was shown that Keap1 controlled degradation of anti-apoptotic Bcl2 protein and apoptotic cellular death. Dysfunctional or mutant Keap1 in cancer cells leads to an accumulation of Bcl2. Moreover, decreased apoptotic cell death and increased cancer cell survival (29). 

These studies indicated that the release of Bcl2 from keap1 and Nrf2-mediated up-regulation of Bcl2, lead to significant accumulation of Bcl2. Increased levels of Bcl2 reduced apoptosis and increased cell survival and drug resistance.

Although age by itself can be the most important prognostic factor in AML patients, cytogenetics and other biological changes could be together with age impact on treatment efficacy (30). Our study results showed that age has an effect on the expression of Bcl2, Bcl-XL, Bax, Nrf2 and Keap1 genes. Data showed that the expression of these genes, in older patients were higher than younger patients. This could result in effective treatment in younger patients. According to our results were not observed a significant difference between gene expression and gender. The overexpression of some genes may be one of the important reasons for ineffective treatment outcomes in older AML patients that cause these patients to need intensive chemotherapy. However, appropriate treatment response, survival and relapse in older AML patients depend on several factors.

Despite modern treatments, recurrence occurred in leukemia patients which may be accompanied with very poor treatment outcomes. Recent findings were indicated that early relapse associated with increased expression of genes involved in proliferation and cancer cells survival (31). Increased levels of Nrf2, Bcl2 and Bcl-XL genes in relapse of the disease might lead to stronger resistance to treatment than the early period of the disease. In recurrence patients (REC) the Nrf2, Bcl2 and Bcl-XL genes expression were higher than NC patients but they were less than CHT patients. These findings show that Nrf2 gene expression is highly increased relative to the new case, but an expression of Bcl2 and Bcl-XL genes were not significantly increased. The relapse in some leukemia patients may occur due to overexpression of cytoprotective, antiapoptotic and oncogenic genes. Actually, the interactions of several molecules lead to the reduction of apoptosis cell, increasing the survival of the cancer cell, chemoresistance and relapse of disease.

**Table 1:** AML Patients' demographic information

Number	Age, years	Sex	WHO diagnosis	Location, City	Treatment Status
#1	28	F	AML with RUNX1-RUNX1T1	Ahvaz	C
#2	6	M	AML without maturation	Behbahan*	N
#3	10	F	AML without maturation	Ahvaz	C
#4	46	M	AML with maturation	Ahvaz	C
#5	22	F	AML without maturation	Ahvaz	C
#6	55	F	AML with maturation	Ahvaz	C
#7	49	M	AML without maturation	Haftkel*	C
#8	16	M	AML without maturation	Ahvaz	N
#9	58	M	AML with maturation	Izeh*	N
#10	26	F	AML without maturation	Ahvaz	C
#11	31	M	AML with myelodysplasia	Ahvaz	C
#12	40	M	AML without maturation	Ahvaz	C
#13	10	F	AML with myelodysplasia	Ahvaz	C
#14	56	M	AML without maturation	Ahvaz	C
#15	23	F	AML with RUNX1-RUNX1T1	Ahvaz	R
#16	47	M	AML with maturation	Aghajari	C
#17	14	F	AML without maturation	Bagh-e Malek*	C
#18	32	F	AML with maturation	Ahvaz	R
#19	43	M	AML without maturation	Ahvaz	C
#20	7	F	AML with minimal differentiation	Dezful*	N
#21	30	F	AML without maturation	Ahvaz	C
#22	20	M	AML with myelodysplasia	Ahvaz	C
#23	57	F	AML with myelodysplasia	Mahshahr*	C
#24	21	F	Therapy-related myeloid neoplasms	Ahvaz	C
#25	44	F	AML with myelodysplasia	Ahvaz	N
#26	38	M	AML with maturation	Ahvaz	C
#27	25	F	AML without maturation	Yasuj**	R
#28	35	F	AML with minimal differentiation	Dezful*	C
#29	9	M	AML with myelodysplasia	Ahvaz	C
#30	58	M	Acute monoblastic/monocytic leukemia	Ahvaz	R
#31	48	F	AML with RUNX1-RUNX1T1	Ahvaz	C
#32	18	F	Acute myelomonocytic leukemia	Ahvaz	C
#33	27	M	AML with maturation	Mahshahr*	C
#34	55	M	Therapy-related myeloid neoplasms	Ahvaz	C
#35	46	M	AML without maturation	Omidiyeh*	C
#36	29	M	AML with myelodysplasia	Ahvaz	C
#37	37	F	AML with minimal differentiation	Omidiyeh*	C
#38	8	F	AML with RUNX1-RUNX1T1	Ahvaz	C
#39	9	M	AML with myelodysplasia	Ahvaz	N
#40	**24**	**M**	**AML with maturation**	Ramhormoz*	**C**

Demographic data in AML patients based on the WHO diagnosis;

* The Patient's home, is in other cities of Khuzestan province.

** The patient's home is in another province.

**Table 2:** The sequences of primers

**Primer length**	**Product (bp)**	**Sequence**	**Primer**
21	172	TGAGCTGGAAAAACAGAAAAA	*NRF2* F
18	172	AATGTCTGCGCCAAAAGC	*NRF2* R
18	150	ATCGGCATCGCCAACTTC	*KEAP1 *F
18	150	CCGGCTGATGAGGGTCAC	*KEAP1* R
20	180	ATGTGTGTGGAGAGCGTCAA	*BCL2* F
21	180	TTCAGAGACAGCCAGGAGAAA	*BCL2* R
20	152	CTGAATCGGAGATGGAGACC	*BCL-XL *F
20	152	CCTCAGCGCTTGCTTTACTG	*BCL-XL *R
20	170	TGC TTC AGG GTT TCA TCCAG	*BAX* F
18	170	GGC GGC AAT CAT CCT CTG	*BAX* R
20	150	GCATCTTCTTTTGCGTCGCC	GAPDH F
22	150	GTCATTGATGGCAACAATATCC	GAPDH R

**Table 3:** Relative expression of Nrf2, KeaP1 genes in AML patients with different age groups. Data expressed as Mean ± SEM

**Age Groups**	**No. of Patients**	***NRF2*** **expression**	**P**_value_	***KEAP1*** **expression**	**P-value**
1-10	7	3.2 ± 1.18	0.32	2.7 ± 1.1	0.423
10-20	3	4.1 ± 1.58	0.201	1.7 ± 0.89	0.651
20-30	14	11 ± 5.24	0.085	1.04 ± 0.73	0.142
>30	16	14.8 ± 8.93	0.146	0.95 ± 0.09	0.265

**Table 4:** Relative expression of Bcl2, Bcl-XL and Bax genes in AML patients with different age groups. Data expressed as Mean ± SEM

**Age Groups**	**No. ofPatients**	**BCL2Expression**	**P-value**	**BCL-XLExpression**	**P-value**	**BAXExpression**	**P-value**
1-10	7	2.55 ± 1.21	0.082	5.23 ± 2.41	0.232	1.9 ± 0.89	0.347
10-20	3	3.44 ± 1.65	0.192	6.41 ± 4.13	0.451	1.2 ± 0.72	0.650
20-30	14	9.35 ± 6.74	0.214	11.67 ± 5.01	0.145	0.83 ± 0.21	0.170
>30	16	10.21 ± 7.53	0.351	12.93 ± 8.11	0.449	0.65 ± 0.09	0.300

**Table 5:** Correlation analysis of relative expression of Nrf2 gene to Bcl2, Bcl-xl and Bax

**Relative Gene Expression**		**BCL2**	**BCL-XL**	**Bax**
*Nrf2*	R	0.000	0.000	0.000
	P	0.673*	0.526*	0.481*

Note: R = correlation coefficient, P = probability.

* Correlation is significant at the 0.01 level (2-tailed).

## Conclusion

In this study, it has been shown that the Nrf2 gene was extremely expressed in various stages of the disease especially in patients treated with chemotherapy. Nrf2 Overexpression dysregulates the expression of genes including antiapoptotic genes like Bcl2 and Bcl-XL. This process results in a reduction of apoptosis, cell survival, proliferation and chemoresistance. Due to the prevalence of Nrf2/Keap1 mutations or overexpression of Nrf2 in a variety of tumors, it seems that inhibition of Nrf2 may be more effective than targeting any target genes on Nrf2 alone. Because of hyperactivation and overexpression of Nrf2 in leukemia, suggest that Nrf2 inhibitors could be used as a pharmacological target in combination with classical chemotherapeutic agents to increase the efficacy of anticancer therapy.
